# Safe and Sound? Scientists’ Understandings of Public Engagement in Emerging Biotechnologies

**DOI:** 10.1371/journal.pone.0145033

**Published:** 2015-12-14

**Authors:** Matthias Braun, Johannes Starkbaum, Peter Dabrock

**Affiliations:** 1 Department of Systematic Theology II (Ethics), Friedrich-Alexander-University Erlangen-Nuremberg, Erlangen, Germany; 2 Department of Sociology, University of Vienna, Vienna, Austria; University Paris South, FRANCE

## Abstract

Science communication is a widely debated issue, particularly in the field of biotechnology. However, the views on the interface between science and society held by scientists who work in the field of emerging biotechnologies are currently insufficiently explored. Therefore filling this gap is one of the urgent desiderata in the further development of a dialogue-oriented model of science-public interaction. Against this background, this article addresses two main questions: (1) How do the persons who work in the field of science perceive the public and its involvement in science? (2) What preferred modes of communication are stressed by those scientists? This research is based on a set of interviews with full professors from the field of biotechnology with a special focus on synthetic biology. The results show that scientists perceive the public as holding a primarily risk-focused view of science. On the one hand, different forms of science communication are thereby either seen as a chance to improve the public acceptance of science in general and one field of research in particular. On the other hand, the exchange with the public is seen as a duty because the whole of society is affected by scientific innovation. Yet, some of the stakeholders’ views discussed here conflict with debates on public engagement in technological innovation.

## Introduction

The relation between science and society is a widely debated issue, particularly in the field of biotechnology. The proclaimed position of policymakers and scholars from the fields of ethics, law, and the social sciences is to move from the previously one-directional and deficit-oriented approach towards a dialogue-oriented mode of science-public interaction [1,2], a development that points to different understandings of the public’s role in scientific innovation.

Pioneer studies from the 1990s have shown that the boundaries between experts and lay publics are fragile, and that different forms of knowledge and expertise exist. Wynne’s work on local understandings of radiation pollution in Northern England, for example, showed that “lay” or “local” people do develop sophisticated (expert) views about science and technology [3]. A famous study by Epstein on the role of gay men in the formerly science-driven debates on AIDS in turn shows how activists acquired credibility through their participation in science [4]. Studies like these have fostered the development of a novel perspective on “lays” and “publics” that acknowledges alternative forms of expertise and therefore favours mutual exchange between scientists, stakeholders and publics. It is for this reason that the increase in public dialogue with and about science can be found on the agenda of several policy papers and recommendations [5,6]. In addition, various scholars have claimed that the modes of public participation in science have become subject to change. It now seems easier than ever for non-professionally trained people to participate in the governance, regulation, and translation of science, as well as in some of the core activities of science itself [7,8]. However it still remains open which concrete form of 'citizen science' will be established in the future and on what grounds. The perspectives range from *citizens as data collectors* to *citizens as ancillary scientists* to *citizens as partners* up to *citizens as full-valued scientists* [8]. Furthermore, scholars from the fields of ethics, law, and the social sciences are currently also debating *who* must and should be involved in the decision-making process when it comes to the further development of science [9,10].

In contemporary practice, widely different models of science communication exist [11]. Brossard and Lewenstein [12] describe four common models of science communication. The deficit model (1), which is undergirded by the idea of a concomitant lack of intellectual understanding and public support for science, is backed up by empirical findings that suggest little scientific literacy and interest within the broader public. The proponents of this model therefore aim to better inform the public about science in order to increase public acceptance. This approach, however, has been widely criticised by, for example, Brian Wynne and Steven Epstein [3,4]. The contextual model (2), on the other hand, acknowledges the heterogeneity of individuals, as well as their capacity to process and further develop given information based on their own experiences and knowledge. Yet this model, too, has been criticised as it nevertheless conceptualizes the public perception of science as a problem that needs to be solved (by experts). The lay expertise model (3) in turn contends that scientists and other elites overestimate their knowledge in relation to public, local, or communal knowledge [13] and maintains that these latter forms of knowledge add valuable information to processes of scientific innovation. Yet the strong focus on local knowledge in this model was also subject to criticism. Finally, the public engagement model (4) aims to enhance public participation in science policy through mediated events such as citizen conferences or deliberative polls. While the basic idea is to democratise science, the resulting focus on the processing or smoothening of science that can often be found in this model has led to criticism as well. To summarise, the first two models favour a one-directional communication mode from the experts towards the public, whereas the latter two embrace the ideals of dialogue and engagement.

In spite of the broad research on science and the public there is only limited knowledge about the preferences of scientists themselves and their understandings of science communication. While a large number of studies have explored public views on (emerging) biotechnologies [13,14,15,16], Bauer and Jensen [17] conclude that there are only a few studies that examine scientists’ views about the public involvement in the governance of science. At the same time, they estimate that a significant number of scientists are already involved in public engagement activities. A report by the Royal Society of London based on approximately 1500 polled scientists and 41 qualitative interviews provides some clearer numbers for the UK [18]. While 74 percent of the respondents reported that they have taken part in public engagement activities, 70 percent also agreed that funders should support these kinds of actions. The interviews showed that the strongest motivator for public engagement is public accountability and the desire to increase the profile of science. Another study by Poliakoff and Webb [19] shows that those scientists who have engaged with the public in the past, those who believe in the significance of public engagement and feel qualified to do it, and those who assume that their colleagues do so as well, are more likely to engage with the public in the future.

In addition, some data exist on scientists’ views about how the interaction with the public is imagined. The 2001 Report of the PABE Project, for instance, is a large study that also involves scientists’ and stakeholders’ views on the public [13]. This report shows how stakeholders tend to describe laypeople as ignoring scientific facts and demanding innovation without risks. The authors of the report challenge these stakeholders’ views, particularly for overvaluing their own perceptions on Genetically Modified Organisms (GMOs). Their focus groups indeed revealed that publics are well aware of the risks associated with GMOs and rather favour a benefit-risk balance. Group discussions with lab staff also showed that one-way communication approaches are dominant among scientists and that communication with the public is repeatedly framed as difficult [20]. Yet this study also identifies narratives by scientists that embrace dialogue with the public. In a recent quantitative study by the Pew Research Center [21] 84 percent of the polled scientists asserted that limited knowledge about science on the side of the public is a hindrance to its acceptance. Finally, a broad study by the German Institut für Demoskopie Allensbach and the Leopoldina German National Academy of Sciences [22] provides some further insights based on a representative poll among the German public, 23 qualitative interviews with scientists, as well as a poll among scientists (p = 106) and journalists (p = 103). A majority of all polled scientists rated “addressing the public with information and communication” as important and self-evident because (a) scientific innovation affects the public, (b) science is heavily funded by public money, and (c) public concerns affect the degree of freedom in scientific research as well as its general framework and conditions, particularly in those areas that receive considerable public attention. They were also confident that better communication about science would increase public acceptance. It was further emphasised that neutrality and objectivity should be the guiding principles, which means that both the benefits and the risks of a certain technology should be made transparent. While communication with the public was generally endorsed, most scientists also said that they have limited options and incentives for addressing the public themselves and that they saw this task to rather lie with journalists and other actors. These studies show that scientists seem to follow different modes of science communication and that the deficit model has not lost much significance so far.

To sum up, there are a number of statements by policy agencies [6,23,24], civil society organisations [25], as well as scientific institutions and associations [5] that provide evidence for the various approaches towards the fostering of new concepts for the mapping and shaping of the interface between science and society. However, the views on the interface between science and society held by scientists who work in the field of emerging biotechnologies are currently insufficiently explored. Therefore filling this gap is one of the urgent desiderata in the further development of a dialogue-oriented model of science-public interaction.

Against this background, this article addresses two main questions: If we acknowledge the widespread demand for the fostering of different forms of public engagement with and within science, (1) how do the persons who work in the field of science think about science communication? (2) How do those understandings relate to the different theoretical models of science communication? In order to answer these questions this article is based on interviews with scientific experts (full professors) from the field of biotechnology with a special focus on synthetic biology.

## Material and Methods

We interviewed twelve scientific experts across Germany working in the field of emerging biotechnologies in general and with a focus on synthetic biology in particular. All interview partners were full professors and internationally recognised for their work.

Due to the selective sample criteria, the scientific stakeholders were purposively selected based on their international reputation and recruited via email. They are located in various regions of Germany, from both former Eastern and Western areas. When asked, they sorted themselves in categories such as genetics, molecular and micro science, chemical engineering or biophysics. Only three scientists did not accept our invitation. The authors of the study recruited and contacted the interviewees themselves and therefore had access to all personal information. Double-blind systems are unusual for the research phases involving these kinds of expert interview studies. Also for the Qualitative Structured Content Analysis, anonymised data is not typical. However, any information provided within the article is anonymous and contains no possibility for tracking single interviewees. The participation in this inquiry was voluntary and unpaid. Informed consent for the participation in the interview study was obtained in writing from all the participants before the beginning of the interviews. An oral consultation with the office of the ethics committee of the Friedrich-Alexander University Erlangen-Nuremberg confirmed that with regard to the voluntary and unpaid participation of the experts no specific consent form is needed.

The interviews were planned as semi-structured expert interviews, an open form of interviewing with a structured topic guide [26]. The question of what actually constitutes an ‘expert’ is, of course, the subject of a larger debate. Bogner et al. [27] stress the fact that scientists and engineers are currently rethinking the factors that define an expert in relation to political decision-making. Within these thought processes it is increasingly acknowledged that multiple actors influence and shape regulative processes. Thematic expertise is, according to this, not necessarily associated with executive power. The scientists interviewed in this project distinguish themselves through their particular knowledge and experience within the fields of biotechnology and synthetic biology.

The applied methodology was also influenced by the problem-centred interview which combines a set of questions on a specific topic to account for both comparability and openness [28]. While the interviews were focused on pre-existing topics, the interviewer aimed to keep a continuing narrative process. The interviews lasted between 30 minutes and one hour. They were either conducted by one social scientist or one ethicist both of whom have the thematic and scientific competence to conduct a productive interview about the specialised field of biotechnology. The interviews were structured into three cluster. The specific questions depended on the issues that the experts themselves raised. Each of the interviews started with some general questions about the respective fields of work of the interviewees, their publication and communication strategies, as well as their ideas on how science and society could and should interact. The second cluster dealt with the scientists’ own perceptions on the impact of their work on society. As such, the interviewees where asked about their professional evaluation of the (possible) impacts of synthetic biology (e.g.: "If you think about your work—or about the work of colleagues from the field of emerging biotechnologies—what kinds of impacts could the findings or newly developed products have?"). After discussing the specific working situation of each interview partner, they were asked about the epistemic constitution of scientific objects that originate from synthetic biology (e.g.: “You said you work with XY. How do you designate them? Are they objects, things, or life forms to you? In how far do you report unexpected or even ‘false’ findings?"). The final cluster addressed the question of the regulation and governance of science (e.g.: "Scientific research is bound to legal frameworks. What impacts do these frameworks have on your work? What role do economic interests play in your research").

The data was audio recorded and transcribed for further analysis. Our major interest was to explore the life worlds of the interviewees [29]. Right from the beginning of the analysis, the interviewers were sensitive towards the research context of the interviewed persons in order to assess their expert statements [26]. In other words, the statements were not analysed as objective realities, but as statements and viewpoints of persons who work in a particular field and therefore have specific forms of knowledge. In another words, this study aims to display the views of scientists without judging them either right or wrong. In our discussion of the interviews, however, we contrast these views with findings from comparable studies as well as studies from the field of public engagement.

The actual analysis was done by qualitative coding, following the techniques of the Qualitative Structured Content Analysis [30,31]. The codes were assigned using a qualitative data analysis software. The code structure was in parts developed as a deductive code based on the topic guide and then inductively complemented with regards to the data. All codes were built on comparable levels of abstraction in order to increase the reliability, yet with enough open induction for producing valid findings on the perspectives of the interviewed. Amongst others, the code categories covered impacts that were described by the interviewees as either positive, negative, or neutral but also strategies, consequences, actors, and particular technologies that were mentioned. Selective parts were analysed interpretively, in order to assess the wider context and the latent structures of meaning [32]. Central phenomena such as public engagement were thus contextualised with other phenomena, the larger structures of meaning, and the things that have not been explicitly said. The following section will describe the narratives about the public-science relationship that could be identified.

## Results

While the interviewed scientists were generally appreciative of the anticipated benefits from emerging biotechnologies, potential side effects and uncertainties of scientific innovation were also identified. The public’s perspective on science on the other hand was imagined as different and more risk-focused (chapter 3.1.). This critical public view is imagined to have an impact on scientific practice and thus necessitates science communication (chapter 3.2.). We distinguished three modes of science communication stressed by the interviewed scientists that contain top-down as well as dialogical logics of communication and engagement (chapter 3.3.).

### 3.1 Anticipated perspectives of the public

When talking about 'the public', the interviewed scientists mainly refer to this entity as a normative and yet not exclusively factual body which exercises control over different sectors of society. This image of the public is typically associated with either the mass media or an undefined number of citizens (general public). Either way, the public is anticipated as an entity that is believed to have a certain image of science and emerging biotechnologies. This image is believed to be a critical one and quite more risk-focused than the scientists’ own perspective on their field of work. The scientist’ stance was mainly explained by two trends: (a) the general public’s lack of knowledge and (b) select publics that monopolise public attention. While the first aspect refers mainly to the imagined general public, the second aspect addresses mostly civil associations and mass media content.

ad a) The general public was often described as having a critical and risk-focused image of science. The interviewed scientists arbitrarily associated this image with a presumed lack of knowledge and insight. Members of the general public were thereby said to base their judgements about science on non-rational facts. While the scientists expressed understanding for public concerns about the future impacts of some high-risk emerging biotechnologies, they rejected the generalisation of all biotechnologies as risky. The scientists criticised that various biotechnologies and techniques are often subsumed under one label and framed as potentially harmful. For the most part, the interviewed scientists perceived their own work in the field of biotechnology as harmless, at least in comparison to other areas. They stressed the necessity of evaluating each case individually instead of lumping them all together.

*"The problem at this stage is that one has to evaluate case-by-case*. *That's why you cannot provide general statements*. *(…) It is clear*, *that if you develop new procedures*, *it is not 100 percent certain what the results will be and which impacts these results will have*. *(…) That's why one cannot say genetics are dangerous or not dangerous*. *Some constructs are completely neutral*, *others might bear problems*, *particularly if they hold pathogens*. *(…) One has to be careful and perform impact assessments*.*" (Interview 3)*

Beyond the assumption that the observed scepticism in society towards different areas of biotechnological research is triggered by risk and security issues, most of the scientists also pointed out that there must be additional reasons. They related that in their experience certain biotechnological developments seem to challenge the beliefs and comprehensive doctrines of a variety of societal actors. In other words, the interviewed scientists asserted that apart from matters about security and risks, challenges to norms and values, such as the rupturing of ethical boarders or the blurring of boundaries between what is defined as natural and non-natural, might heavily shape public views on emerging biotechnologies. From a scientific perspective, these public viewpoints are not always rational. The interviewees stressed for example that natural evolution alters genetic codes even more than simple engineering efforts in laboratories. The scientists often expressed a non-essentialist approach to scientific products and compared the results of their work with entities that appear in the 'natural world'. This argumentation was backed up by the description of assumed natural entities, such as agricultural crops, that have been repeatedly crossed with other plants and said to have only little in common with its origin archetype. The following statement recounts an encounter with a journalist who brought up the metaphor of 'playing God' in science.

*"This reminds me of a conversation I had with a journalist*. *We were standing in front of a corn plant and the interviewer said*: *why do you do genetics and why do you think that you can make this plant better than God*? *Then I asked him*: *Do you think God has made this plant*? *Corn is an artificial product made by humans*. *Humans have*, *over thousands of years*, *developed a small plant with small corns into this one here*. *This has little to do with God*. *If it was God*, *then it was because he wanted it*, *because he gave us the opportunities to do so*. *We got a brain and we should use it*. *And if we had not bred plants we would still be hunters and gatherers*.*" (Interview 2)*

While scientists indeed expressed understanding for the anticipated viewpoints of the general public, public views were nevertheless often perceived as biased.

ad b) During the interviews, scientists formulated criticism on the selective image of science communicated by the mass media and some critical civil associations. Even though all interviewed persons expressed an implicit awareness of the risks and possible hazards associated with their work, they also decried that select groups monopolise and exaggerate these topics and their appraisal in the public (media) discourse. This position is based upon the understanding that public discourse is not always representative of the opinions of all citizens within a specific space, but rather of those opinions of smaller groups that monopolise the attention in the media or other protest formats. In response to these cases, the interviewed scientists advocated for the democratisation of public discourse for the sake of the inclusion of the general public as they were confident that the views of the general public are comparably less critical towards science.

*"I find it important that we take care of finding a balanced consideration of different interests and risks*. *I think it is an indefensible situation that interest groups can and do dominate whole societies*, *as it was the case with green biotech in Germany*. *The actual guidelines [of those interest groups] are based only to a limited extent on serious facts*, *but mostly on diffuse scenarios of fear that are produced by interest groups specifically for this topic*. *They cannot be proven by rational facts*. *Please don't get me wrong*, *I sincerely believe that scientists*, *like all specialists*, *urgently need correctives from the outside*, *to see the consequences of their work*. *Yet*, *I become angry if I get the feeling that I am being exploited in order to support the power position of someone else*.*" (Interview 6)*

Besides these perceptions on the public, the interviewed scientists randomly referred to impacts associated with these publics. It will be shown later that these impacts explain, to some extent, the scientists’ preferred modes of science communication.

### 3.2. Impacts for scientific practice

The previous chapter showed that the interviewees perceive the public as critical towards science. At the same time, scientists described their field of work as increasingly transparent for the public and other stakeholders. The public in turn was constructed as an entity that is able to impact or even control science. The two most commonly named impacts for scientific practice, which were also related to each other, were (a) an increase in control and regulation initiated by public attention and action and (b) the necessity as well as new possibilities for the promotion and fostering of research.

ad a) Emerging biotechnologies were repeatedly described as increasingly transparent and under public control—as deduced from, for example, the mass media or the results of opinion polls. Public preferences were perceived as impacting policies on science and can therefore initiate regulative elements or even the prohibition of certain forms of research, such as stem cell research with foetuses in Germany. While all interviewed scientists emphasised the importance of external control mechanisms such as laws or security levels, some control mechanisms were criticised as too strict and as hindering innovation. Science was also perceived as opening up through new forms of public involvement such as citizen science, which relates to persons from the general public who actively involve themselves in science. Such an increasing demand for more public participation challenges existing ideas of what constitutes good scientific practice and raises questions about new forms of expertise.

This perceived increase in the transparency of science was accompanied with the observation that certain fields of science gain comparably more attention than others. By way of example, media reports were identified as repeatedly focusing on high-risk cases while ignoring those cases that are more significant for many biotechnologists (but that are predominantly assessed as having only low levels of risk potential). This selective image was said to produce a biased image of science which, yet again, limits the general public’s potential insights into science. A selective emphasis on certain types of science was also said to affect funding options, an aspect which already relates to the next impact type.

ad b) The interviewed scientists related how public transparency also leads to a necessity as well as new possibilities for the promotion and fostering of research. Once fields of research, such as Synthetic Biology, enter into the public discourse, they might increase the funding and work options for scientists. This was said to be particularly the case when societal benefits become part of the public debate. Promoting one's field of work can therefore be beneficial for scientists, since it might lead to an increase in funding options and political support. This viewpoint relates to the expressed confidence in scientific benefits and the related belief that communication is likely to increase public acceptance. Some scientists explicitly referred to Drew Endy, a well-known scientist who works in the field of synthetic biology and repeatedly appears in the media discourse on science. One of the interviewed scientist claimed that scientists like Drew Endy might promote things they cannot meet but still lay the groundwork for further research by opening up a public debate.

*"Drew Endy*, *you might think about him what you want*, *[…] but he basically has brought this topic [synthetic biology] repeatedly on the table and simply incessantly advertised for it*, *which lead to other people*, *who got further than him*, *getting pushed*. *Such visionaries are necessary*. *I believe that without this showing off–which I myself do not like that much*, *but some people master well—without that*, *no field of science would really progress well*. *If we were all modest it would take very long until something would happen*.*" (Interview 5)*

The image of the public described here, as well as the power over science attributed to it, explains the many narratives about science-public interaction during the interviews. The scientists presented different modes of communication which will be discussed in the following section.

### 3.3. Modes for science-public interaction

The interaction with the general public was a dominant topic during the interviews. It was never questioned that the public should somehow be addressed with communication, but the actual described and preferred forms of science-public interaction differed across and within the interviews.

As outlined above, four ideal typical modes of science-society interaction have been distinguished by Brossard and Levenstein [12]: the deficit oriented mode, the contextual mode, the lay expertise mode, and finally the public engagement mode. The first important result from the interviews is that the deliberations of the experts support but simultaneously amend these ideal modes of science-society interaction. Thus, in the view of the stakeholder, the borders between the different modes are not clearly defined—even within the individual interviews. The second important result is that there were no actual references within the rational deliberation of the stakeholders to the lay expertise mode. Very frequently the stakeholders refer to the deficit mode (1) as well as to the public engagement mode (3). Some deliberative remarks refer to the contextual model as well (2).

ad 1) The deficit mode became visible in the deliberations which favoured a one-directional release of information about research and its aims. Yet in this particular mode information was not intended to enlighten the public in a Habermasian sense [33], it was primarily seen as a way to ease the public and its anxieties. The public is thereby imagined as ignorant and uninformed about science. This ignorance is believed to result in a failure of communication that cannot easily be resolved. A major problem is the inability of the public to understand what scientists actually do.

*„One can treat the public like in kindergarten*: *one explains with building brick models*, *and says it is like that*, *you won’t understand*, *but I’ll explain it to you in very simple terms–this is dismissive in a way*. *You never know what the receiver of the information will think*. *Either he thinks that what they do is easy*, *or he doesn’t know what it is about*. *Anyway–it is always like that–I cannot explain what I am interested in*.*” (Interview 3)*

One solution to this dilemma is thought to lie in creating positive images of science and its impacts. Following this mode, it is not relevant whether this image correlates with scientists' actual understanding of science, but rather that it creates an image of science that is favoured by the public. This relates to a more or less participation driven image of the public, while the scientists describe themselves as dependent on (critical) public judgements.

*„…I don’t want to say false pretences*, *but it is part of it*. *The public expects something useful from scientists–and then you build on that*. *And luckily they don’t ask–have you brought further cancer research*?*–luckily not*. *Therefore it is a silent accordance that scientists can do what they want and once in a while something useful comes out*.*” (Interview 3)*

ad 2) The deficit-contextual mode was observed in statements that comparably favoured a one-directional top-down communication approach in those cases where scientists expressed confidence that once they explain their work from their own perspective, the public might become more accepting of it. Yet members of the public are, according to this mode, not ignorant but largely uninformed and therefore critical towards science–a situation that can be addressed by disclosing more information. In other words, providing information to the public by representatives of scientific research is believed to increase the acceptance of science.

The public is, according to this logic, distracted from the anticipated benefits of science by receiving only limited or selective information (see chapter 3.1).

The very logical consequence of this perception is to make science transparent. It was often expressed that many public expressions of risk have its origin in a lack of insight in the specific actions performed by scientists. According to scientists’ perceptions, the expert knowledge of scientists would allow the public to evaluate biotechnology based on scientific facts. Limited or simplified information was said to result mainly in resistance or unknown reactions towards specific areas of research. Therefore, the scientists who adhere to this belief suggest informing the members of the public so that they can form their own image of science–a strategy that is believed to result in a more positive evaluation.

*"Before every talk I hold on genetics*, *particularly green genetics*, *I say*: *I tell you what a scientist knows about it*. *Then it is the task of the audience to decide whether they take a decision based on what I have said or if they are against it*. *(…) The question is then*, *in how far can a scientist contribute to make sure that a certain viewpoint does not become skewed*. *Because when I look at how green biotech is discussed nowadays*, *it makes my hair stand on end [metaphor for being shocked]*.*" (Interview 4)*

This last quote above indicates the possibility of (uninformed) public protest. Any type of information is believed to be able to trigger this kind of reaction. Nevertheless, providing information about research and public education is still seen as the most reasonable approach within this model. The public is perceived to have the potential to understand science; yet in practice it is believed to lack insight and education, as the following statement demonstrates:

*"For me it seems that a central point is to ensure a high and increasing level of public knowledge about what we know in biology*, *for example through biology classes at schools*. *(…) Because for the evaluation of risks it is of utmost importance to reduce and weaken the knowledge gap between experts and the general public*. *If this knowledge gap is extreme*, *it can lead to an enforced silencing of certain topics*, *or even their demonization*. *A narrower knowledge gap in contrast would contribute to a better and more rational evaluation of risks*, *which would in turn lead to more scientific freedom to further develop the really interesting and important things*.*" (Interview 7)*

ad 3) Once the interviewed scientists referred to a public engagement mode, they stressed that the broader public should either have access to or be engaged in scientific practice. Dialogical engagement is mainly understood as the exchange of information and perceptions. Transparent information, discussion meetings, and comparable formats were typically named in this context. Bottom-up activities such as citizen science were mentioned as well, but only seldom. Once named, the legitimacy of public engagement activities was almost never questioned and repeatedly described as a duty in contemporary democracies. However, most statements within this mode included both top-down and more dialogical logics of communication, such as the following quote by the same scientist demonstrates:

*"I believe that the public has the right to know what we do*. *The public also has the right to hear about what kind of risks will be involved according to the present state of science*. *(…) There should be mechanisms in place to prevent any undesirable developments*. *Something that no longer has public consensus should no longer be supported*. *So much is clear*, *it should be like this in a democracy*. *And these opportunities for intervention must be given (…) for these new technologies*, *they must be discussed at an early stage*.*" (Interview 7)*

To sum up, the interviewed scientists primarily described the public as critical towards science while having the power to impact or even control science. This perception explains the strong demand for different modes of science-public communication.

## Discussion and Conclusion

At the beginning of this article we stated that science communication is on the agenda of recommendations by various stakeholders. Furthermore, previous studies have shown that scientists are tendentially open to science communication [17,34]. When addressed in debates about public engagement, the role of scientists is often assumed to be either supportive or passive. Wilsdon and Willis stated that:

"The science community has embraced dialogue and engagement, if not always with enthusiasm, then at least out of a recognition that BSE, GM and other controversies have made it a non-negotiable clause of their ‘licence to operate‴ [34].

However, in practice and theory, there exist different modes of science communication as for example Brossard and Lewenstein [12] have pointed out. Our presented interview data shows that scientists indeed express different motivations and understandings of science communication. Yet, whereas some of these modes correspond with those from literature, other modes from the literature were not found in the scientists’ narratives at all.

This study therefore complements the existing knowledge by providing a detailed view of (1) the conditions that encourage scientists to engage in science communication, as well as (2) different modes for science communication stressed by the interviewed scientists.

ad 1) Former studies have shown that many scientists affirm the necessity of science communication [22]. The motivations for this assertion, however, has so far been mainly explored in general categories, such as 'to increase the profile of science' [18]. The data presented here suggests that this "necessity" is connected to a prevailing image of the public, as well as to the expected impact of the public on scientific practice.

The public is imagined by the interviewed scientists as critical towards science. This corresponds with the quantitative study by the Pew Research Center [21] which already showed that US scientists perceive fewer risks with regards to new technologies than does the general public and that the majority of the scientists identifies limited public knowledge as the major hindrance to the acceptance of science. The PABE report has similarly shown that scientists imagine publics to be risk-focused, that they often ignore scientific rationality, and that they therefore underestimate possible benefits [13]. In the course of the study by the German Institut für Demoskopie Allensbach and the Leopoldina German National Academy of Sciences [22], scientists asserted that public concerns affect the degree of freedom and the general framework and conditions of science.

The interviewed scientists from our study likewise said that the public, and its vision of science, is able to impact scientific practice (see chapter 3.2.). Public perceptions were perceived as relevant for policy makers and funding agencies that govern science. Additionally, the trend of some members of the public actively engaging as "citizen scientists" was mentioned as a factor of influence for science. On the one hand, the increased public attention for science was welcomed as an external control factor for science, but on the other hand it was also seen as a problem due to the perceived critical stance of the public. Scientists therefore described the necessity to communicate or even to promote their field of work. Our study has shown that there exist different understandings of science communication in the narratives of scientists, yet they do not fully correspond with those modes described in the literature [12].

ad 2) Science communication is a dominant topic in policy reports, in other literature, and also in the presented interview data. Former studies have provided a first glimpse into scientists’ understandings of science communication. The study by the German Institut für Demoskopie Allensbach and the Leopoldina German National Academy of Sciences [22] has shown that scientists evaluate communication and information as important since they see themselves as being in an interdependent relationship. The polled scientists in this study expressed the confident belief that neutral and objective information about science will increase public acceptance. Other studies on scientists' views concluded that a deficit approach is dominant, though dialogue-oriented approaches are also found [13, 20, 21]. While some studies suggest that a reasonable number of scientists have taken part in public engagement activities [17, 18, 19], other studies highlight that scientists lack time and incentives to engage themselves in it and regard it as the task of other actors [22].

The interview data presented here shows a comparable image to those studies cited above, yet it adds further insights into which concrete modes of science communication scientists prefer, and it shows which modes they do not perceive as important. The interviewed scientists preferred different forms of science communication that could be related to some of the modes described by Brossard and Lewenstein [12]: deficit, deficit-contextual, and public engagement. Whereas the first two favour a top-down mode of communication towards publics the last one builds on a dialogue-oriented approach.

The deficit and deficit-contextual modes of science communication equally build on the idea of improving the public acceptance of science by a top-down transmission of information. The difference between those two models, however, relates to the imagined role and the perceived capacities of the public. Whereas the first assumes the public to be somewhat ignorant and manipulable, the second sees the potential of the public to recognise the scientists' perspective of science. Both viewpoints assume that public views have the tendency to underestimate the benefits from emerging biotechnologies and that closing the knowledge gap between the general public and scientists may result in a greater acceptance of science

However, addressing the public was not imperatively understood in a top-down manner during the interviews, nor in other studies in the past [20, 22]. Once a public engagement mode was in place, scientists favoured a dialogue-oriented form of communication. The public was thereby conceived of as the recipient of the benefits as well as the risks associated with scientific innovation, thus addressing the public in dialogue was emphasised as a reasonable and democratic practice. This perspective is less concerned with anticipated the deficits in public discourse and more with the normative understanding of opening science up to a broader public and fostering a mutual understanding. Yet whenever this mode was promoted, the scientists typically combined it with aspects from the one-directional modes of communication.

In summary, scientists expressed interest in and the necessity for addressing and including the public in scientific innovation–yet in doing so they each expressed their own understanding of the role of the public. The science communication modes described by the interviewed scientists differ from the modes found by Brossard and Levenstein [12]. The most fundamental difference was that there were no narratives that followed the logic of the lay expertise mode. This means that many of the scientists were not very confident about the benefits to be gained from including lay knowledge in the governance of science. They merely perceived the public as an external entity of control rather than as an active participant in the production of a constructive impact. However, theory, policy recommendations, and studies of public engagement tell a different, a more inclusive story [1,2]. Awareness of these different viewpoints and expectations of science communication and public engagement is valuable for the organisation of public engagement in practice.

So far, efforts by scientific scholars, policy makers, and other stakeholders to understand the relationship between science and society have placed much more attention on public viewpoints–as publics have indeed historically been excluded from the governance of science. Nevertheless, the role of scientists in this new trend is still marginalised. If the widely heralded spirit of responsible research and innovation [35] is to be taken seriously, the views and conditions of all stakeholders must be taken into account when the governance of science is subject to change. The presented data suggests that it might be worthwhile to think about systematically improving the opportunities and institutional incentives for the inclusion of scientists in public engagement activities where they can exchange their views with those of citizens.

## Appendix

### Original interview quotes in German

*"Ich glaube auch*, *dass wir ohne die Gentechnik (…) kaum gute Chancen haben*, *uns zu wappnen für die Zukunft*. *Bei der Roten sehen wir das schon—fast alle Medikamente sind rekombinant hergestellt*. *Das ist einfach ein Fakt*. *Deutschland war da ganz vorne dabei*, *dann wurde aber die Produktion von rekombinantem Insulin zum Beispiel verboten*, *weshalb diese Art der Arbeiten eingestellt wurde und jetzt importieren wir das alles*.*" (Interview 2)*

*"Aber ich denke*, *(…) synthetischen Viren–dass man da in Grenzbereiche kommt*, *die man halt irgendwie–wenn sie mal draußen sind*, *kann man sie nicht mehr einfangen*. *Und man kann nicht jeden Effekt prognostizieren*.*" (Interview 9)*

*"Das erinnert mich so an ein Gespräch*, *hatte ich mal mit einem Journalisten*, *da standen wir vor Maispflanzen und der interviewte mich dann*: *Ja*, *wieso machen Sie denn Gentechnik und wieso glauben Sie denn*, *dass Sie die Pflanzen da besser machen können als Gott*? *Dann habe ich ihn gefragt*: *Glauben Sie*, *die Pflanzen hat Gott gemacht*? *Mais ist ein Kunstprodukt von Menschen*. *Also*, *der Mensch hat über zigtausend Jahre aus einer Pflanze*, *die ganz kleine Körnchen hatte*, *ganz klein war*, *haben sie diese Pflanzen erzeugt*. *Das hat mit Gott an der Stelle wirklich nicht viel zu tun*. *Wenn es mit Gott war*, *dann*, *weil er es wollte vielleicht*, *weil er uns die Möglichkeit gegeben hat*, *das zu tun*. *Wir haben Hirn bekommen und wir sollen es auch benutzen*. *Und wenn die Züchtung nicht stattgefunden hätte*, *dann würden wir immer noch Jäger und Sammler sein" (Interview 2)*

*"Also*, *das Problem an dieser Stelle ist*, *man muss eine Fall-für-Fall-Betrachtung anfangen*. *Und darum kann man kann keine generellen Aussagen machen*. *(…) Also*, *ich meine*, *es ist klar*, *dass man*, *wenn man neue Verfahren entwickelt*, *ist es nicht hundert prozentig sicher*, *was man im Prinzip dann als Ergebnis bekommt und welche Auswirkungen diese Ergebnisse haben*. *(…) Und darum kann man auch nicht sagen*, *Gentechnik ist gefährlich oder ist nicht gefährlich*. *Manche Konstrukte sind komplett neutral*, *andere Produkte können Probleme beinhalten*, *ganz speziell*, *wenn sie pathogene Gene beinhalten*. *(…) Man muss einfach vorsichtig sein*, *muss Technikfolgenabschätzung im Prinzip dann machen" (Interview 3)*

*"Wichtig finde ich*, *dass wir dafür sorgen*, *dass eine ausgewogen Berücksichtigung aller Interessen und Risiken gefunden wird*. *Ich halte es zum Beispiel für einen unhaltbaren Zustand*, *das Interessengruppen eine ganze Gesellschaft dominieren dürfen und können*, *wie wir es in Deutschland bei der Beurteilung der Grünen Gentechnik erfahren haben*. *Die aktuellen Regularien beruhen nur zu einem geringen Maße auf ernstzunehmenden Fakten*, *sondern maßgeblich auf diffusen Angstszenarien*, *die durch die entsprechenden Interessengruppen speziell für dieses Thema aufgebaut werden*, *die sich aber nach wie vor nicht rational belegen lassen*. *Bitte verstehen Sie mich nicht falsch*, *ich glaube fest daran*, *dass Wissenschaftler*, *wie wohl alle Spezialisten*, *dringend Korrektive von außen brauchen*, *um die Konsequenzen ihrer Arbeit absehen zu können*. *Ich werde aber immer ärgerlich*, *wenn ich das Gefühl haben muss*, *instrumentalisiert zu werden für die Stützung von Machtpositionen von irgendjemandem*.*" (Interview 6)*

*"Ich sage vor jedem Vortrag*, *den ich halte*, *wenn es Richtung Gentechnik geht*, *ganz speziell grüne Gentechnik*: *„Ich erzähle hier jetzt*, *was ein Wissenschaftler darüber weiß*.*”Und dann ist es eine Aufgabe der Zuhörerschaft*, *zu entscheiden*, *ja*, *ob auf der Grundlage*, *was ich vermittelt habe*, *ja–dann eine Entscheidung trifft*, *ob sie dafür oder dagegen sind*. *(…) Und die Frage ist eben*, *inwieweit kann der Naturwissenschaftler dazu beitragen*, *dass eine bestimmte Einschätzung dann nicht in eine total verquerte Richtung läuft*. *Denn wenn ich heutzutage mir anschaue*, *wie grüne Gentechnik öffentlich diskutiert wird*, *dann stehen einem die Haare zu Berge*.*" (Interview 4)*

*"Und ein ganz wichtiger Punkt dabei erscheint mir aber zu sein*, *dass man dafür Sorge trägt*, *zum Beispiel im Rahmen des Biologieunterrichts an den Schulen*, *dass es einen hohen und wachsenden Kenntnisstand der Bevölkerung gibt über das*, *was man in der Biologie weiß*. *(…) Denn bei der Beurteilung der Risiken ist es ganz wichtig*, *das Kenntnisgefälle zwischen den Experten und der allgemeinen Bevölkerung abzuschwächen*, *zu verringern*. *Dieses Kenntnisgefälle*, *wenn das eben extrem ist*, *kann das zu einer schnellen Tabuisierung führen von Dingen oder Dämonisierung sogar*, *während eben ein flaches Kenntnisgefälle mit dazu beiträgt*, *dass Risiken besser eingeschätzt werden*, *rationaler eingeschätzt werden und damit auch wiederum die Wissenschaft mehr Freiräume bekommen kann*, *um die wirklich interessanten und wichtigen Dinge weiterentwickeln zu dürfen*.*" (Interview 7)*

*"Ich denke*, *dass die Öffentlichkeit Anspruch hat darauf*, *zu wissen*, *was wir dort tun*. *Die Öffentlichkeit hat auch Anspruch darauf*, *zu erfahren*, *welche Risiken damit nach dem aktuellen Kenntnisstand der Wissenschaft verbunden sein werden*. *(…) es muss Instrumentarien geben*, *um totale Fehlentwicklungen wirklich dann zu unterbinden*. *Etwas*, *was gesellschaftlich keinen Konsens mehr hat*, *darf dann nicht mehr unterstützt werden*. *Das ist klar*, *in der Demokratie muss das so sein*. *Und diese Eingriffsmöglichkeiten muss es (…) jetzt bei diesen neuen Technologien–muss das frühzeitig diskutiert werden*.*" (Interview 7)*

*"Ich meine*, *Drew Endy*, *von dem mag man halten*, *was man will*, *[…] aber der hat halt im Grunde dieses Thema [Synthetic Biology] immer wieder aufgebracht und hat einfach wahnsinnig viel Werbung dafür gemacht*, *sodass auch Leute*, *die jetzt viel weiter kommen als er*, *sozusagen angeschoben wurden*. *Und so Vordenker braucht es*. *Also*, *ich glaube schon*, *dass*, *also ohne diese Schaumschlagerei*, *die mir selber jetzt nicht so liegt*, *aber die eben manche Leute sehr gut beherrschen*, *ohne die kommt wahrscheinlich keine Wissenschaftsrichtung so richtig voran*. *Also*, *wenn wir alle nur so bescheiden sind*, *dann dauert es sehr lang*, *bis irgendwas vorangeht*.*" (Interview 5)*

*„Man kann die Öffentlichkeit so ins Kindergartenalter stecken*, *dann erklärt man das so mit Bauklötzchen-Modellen und sagt*, *das ist so und so*, *ihr begreift das sowieso nicht*, *aber ich mache es jetzt mal ganz einfach für euch*, *so herablassend in gewisser Weise*. *Das ist eigentlich der–weiß man immer nicht*, *was der Empfänger dieser Botschaft dann denkt*. *Entweder denkt er*, *das*, *was die da machen*, *ist alles ein Kinderspiel*, *oder worum geht es eigentlich*. *Aber es bleibt immer so*, *also das Eigentliche*, *was mich interessiert*, *kann ich euch gar nicht erklären*.*”(Interview 3)*

*„…ich will nicht sagen*, *eine Vorspiegelung falscher Tatsachen*, *aber das gehört natürlich dazu*, *dass man–die Öffentlichkeit erwartet von den Wissenschaftlern irgendetwas Nützliches*. *Und dann bedient man das so ein bisschen*. *Und zum Glück fragen die ja nicht nach*, *ja*, *wie ist denn das*, *habt Ihr jetzt die Krebsforschung vorangebracht*, *oder so*, *zum Glück nicht*. *Also insofern ist das ein stillschweigendes Einverständnis*, *dass die Wissenschaftler machen*, *was sie machen wollen*. *Und es kommt ja auch ab und zu was Nützliches raus”(Interview 3)*
